# Noise-Reducing Fabric Electrode for ECG Measurement

**DOI:** 10.3390/s21134305

**Published:** 2021-06-23

**Authors:** Takamasa Terada, Masahiro Toyoura, Takahide Sato, Xiaoyang Mao

**Affiliations:** 1 Department of Computer Science and Engineering, University of Yamanashi, Kofu, Yamanashi 400-8511, Japan; g19dts03@yamanashi.ac.jp (T.T.); mao@yamanashi.ac.jp (X.M.); 2 Department of Electrical and Electronic Engineering, University of Yamanashi, Kofu, Yamanashi 400-8511, Japan; takahides@yamanashi.ac.jp

**Keywords:** textile, electrocardiography, denoising

## Abstract

In this work, we propose a fabric electrode with a special structure that can play the role of a noise reduction filter. Fabric electrodes made of the conductive fabric have been used for long-term ECG measurements because of their flexibility and non-invasiveness; however, due to the large impedance between the skin and the fabric electrodes, noise is easily introduced into the ECG signal. In contrast to conventional work, in which chip-type passive elements are glued to the electrode to reduce noise, the proposed electrode can obtain a noise-reduced ECG by changing the structure of fabric. Specifically, the proposed electrode was folded multiple times to form a capacitor with a capacitance of about 3 nF. It is combined with the skin-electrode impedance to form a low-pass filter. In the experiment, we made a prototype of the electrodes and measured ECG at rest and during EMG-induced exercise. As a result, the SNR values at rest and during exercise were improved about 12.02 and 10.29 dB, respectively, compared with the fabric electrode without special structure. In conclusion, we have shown that changing the fabric electrode structure effectively removes noise in ECG measurement.

## 1. Introduction

In the field of e-textiles, conductive yarns, and conductive fabrics are used as sensors. Conductive fabrics have the advantages of being flexible and lightweight compared to conventional electrodes such as metal plates. In addition, conductive fabrics have the advantage of non-invasiveness, so they are also used as electrodes for measuring biological signals, as in electrocardiogram (ECG). However, it is known that fabric electrodes cause the introduction of a great deal of noise because the skin-electrode impedance is higher than with a wet electrode, such as an Ag/AgCl electrode [1]. In general, noise reduction techniques are required in ECG analysis such as the automatic diagnosis of heart disease. If noise reduction is not sufficient, the accuracy of the automatic diagnosis may decrease because the ECG feature extraction accuracy like R-peak detection will also decrease [2]. For noise reduction, various methods have been proposed, such as the improvement of electronic circuits, materials of electrodes, and noise reduction algorithms. In this work, we propose a noise reduction method focusing on the internal structure of the electrode.

Conventional ECG measurement devices use an analog filter that combines flexible materials and passive elements, such as chip resistors and capacitors. For example, one proposed device involves a printed conductive pattern and a passive device attached to the fabric to form an analog filter. This structure makes it possible to acquire a less noisy signal than when using electrodes without a filter. However, there is a risk of damaging the conductive pattern and the passive device by disturbance. To prevent this damage, a new approach that omits the process of attaching the passive device to the fabric and makes the fabric itself function as a circuit is required. Recently, a pressure sensor that can reduce the number of conductors connecting threads and circuits has been proposed [3]. This sensor is focused on the structure of thread intersections, and it has been shown that just changing the structure of the fabric can provide a new function. This means that the passive elements used in the circuits can be replaced by changing the internal fabric structure. In the field of ECG measurement device development, an electrode that changes only the structure of the conductive fabric itself to add function as a filter has not been proposed. Therefore, we propose a new noise reduction method that allows band-limiting by changing only the internal electrode structure without using passive elements, such as chip devices.

In this work, based on the idea in e-textiles of adding functions by changing the structure of conductive yarns, we consider changing the structure of fabric electrodes to add new functions. As a new function, the multi-layer structure of the electrode provides a capacitor which has a sufficient capacitance. Then, the capacitor and the skin-electrode impedance are combined to form a low-pass filter. The contributions of this work are as follows:

- We have discovered that elements made of fabric can play the same role as general passive elements;
- We show that the impedance of the human body and passive devices made of conductive fabric can work as a filter;
- We show that changing the internal structure of the conductive fabric is effective in noise reduction.

In this paper, we show that, by changing the structure of the electrode itself, it functions as a filter. In the experiment, the proposed electrode performs an ECG measurement with little noise even when the electrode is dry. The paper is organized as follows: Section 2 describes related works; Section 3 describes the proposed electrode structure; in Section 4, the ECG measurement system is used to evaluate the proposed electrode; Section 5 describes the discussion and future works; and finally, Section 6 summarizes this work.

## 2. Related Works

Monitoring systems for various activities, including early detection of heart disease, have been proposed [4]. In such system, clean ECG data are required for activity identification. To realize continuous ECG measurements in daily life, various improvement of measurement devices and electrodes have been proposed.

Komensky et al. [5] proposed a method for an active electrode in which a pre-amplifier and an electrode were mounted on a polychlorinated biphenyl (PCB) substrate. This device is a belt-type device that can perform an ECG by being wrapped around the chest. However, it has been pointed out that electrodes using PCBs are hard and may cause discomfort to the wearer. Using flexible instead of hard materials is one way to reduce such discomfort. For example, measurement devices using conductive rubber materials [6,7,8] and graphene [9,10,11] as electrodes have been proposed. Reyes et al. [12] proposed a carbon black powder/PDMS composite electrode, and showed that ECG waveforms could be measured in dry and water immersion, as well as with the Ag/AgCl electrode. Furthermore, flexible materials coated with conductive materials on non-conductive fabrics and yarns coated with conductive materials [13,14] have also been proposed. Taji et al. [15] proposed a wristband device made of silver-coated nylon as a flexible material and showed that ECGs could be performed correctly, even with the fabric electrode. Rattfalt et al. [16] found that differences in electrode materials can change the electrical properties of fabric electrodes, such as impedance. Sneh et al. [17] proposed fabric electrode composited the graphene and PEDOT:PSS which has lower impedance. They showed that it is possible to measure the ECG with high peak amplitude.

It has been reported that not only the material but also the structure of the electrodes affects the quality of the ECG. Wu et al. [18] developed an electrode that provides both comfort during measurement and quality of signal by changing the ratio of silver to cotton yarn. Xiao et al. [19] created a fabric electrode with a honeycomb structure made of silver-coated nylon. This work showed that different proportions of conductive and non-conductive yarns and different weaves affected the quality of ECGs and the wearer’s comfort when the electrodes were attached. However, changing the structure to add functions has not been discussed.

An improvement of the combination of the electrode and the device has also been proposed. To reduce patient discomfort, Li et al. [20] proposed a wearable device in which a band with a thick fabric electrode was wrapped around the chest to ensure sufficient skin-to-electrode contact. Meanwhile, Lee et al. [21] proposed a wearable device using a conductive foam as a material instead of fabric, which allows the skin and the electrode to be in closer contact and reduces low-frequency noise. These devices are designed to place an insulator, such as clothing, between the skin and the electrodes, so there is a capacitor between the electrodes and the skin. By inserting bias resistors from a few MΩ to a few GΩ in parallel with the pre-amplifier, the low-frequency noise can be reduced. However, it has been reported that other noises can be introduced easily depending on the thickness of the clothing. On the other hand, Lee et al. [22] show an example of a low-pass filter that includes a capacitor in parallel before the pre-amplifier. In their device, the capacitance generated between the electrodes and the device shield is small, a few pF, and is treated as parasitic capacitance. Takano et al. [23] proposed an application to bed sheets. They proposed a structure in which capacitance is generated by inserting a shield between the electrode and GND, but without changing the structure of the electrode itself. Ozkan et al. [24] proposed a remote ECG monitoring system in which a conductive fabric was used as an electrode for the device. Their fabric electrodes were removable and washable, but electrodes in their system were just conductive fabric, meaning the electrodes themselves do not include a filter like an active electrode. This implies that, if the fabric itself has a function to reduce noise, the noise rejection performance of the system can be improved.

Conventional fabric electrodes are made of conductive yarn or a non-conductive fabric coated with conductive materials. There are methods to build a high-pass filter using the capacitance between the skin and the electrode and a resistor before the pre-amplifier, but they require a resistor of several MΩ to several GΩ in the electrode. Such a large resistance is difficult to miniaturize for integrated circuits, and chip resistors must be mounted on a conductive fabric. In addition, electrodes using electronic elements such as resistors and capacitors lose the advantages of fabric because they may be damaged by moisture or impact. If these elements are flexible like fabric and do not require adhesion or other treatments to attach them to the fabric, we can make more robust electrodes. We, therefore, propose an electrode with a capacitor by changing the structure of the fabric electrode and show that the skin-electrode impedance forms a low-pass filter. Then we design a first-order low-pass filter to show that the proposed electrode works as a filter.

## 3. An Analog Filter by the Conductive Fabric

In this section, the structure of the proposed fabric filter and the actual fabric electrode are presented.

### 3.1. Requirements for Analog Filter

**Figure 1:**
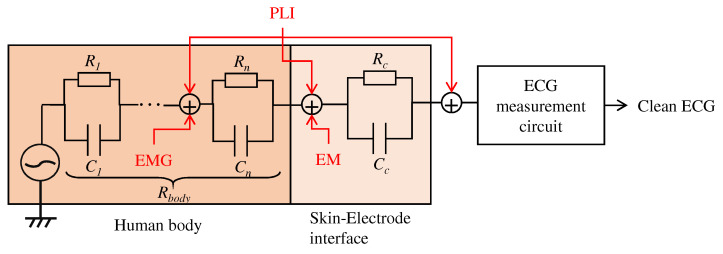
Relationship between the human body, skin-electrode interfaces, ECG measurement system, and noises. PLI, EMG and EM mean power line interference, electromyogram, and electrode motion, respectively.

As described in Section 2, this work aims to create a low-pass filter without an electronic device by changing the structure of the electrodes. We will firstly describe the relationship between the proposed electrodes and the noise to be filtered, then the proposed structure of the fabric. As shown in Figure 1, there are several types of noise, such as power line interference (PLI), electrode motion (EM), and the signal from organs other than ECG, such as electromyogram (EMG). It is known that PLI is 50 or 60 Hz, EM is low frequency, and EMGs are high frequency [2]. Since EMGs are noise originating in the human body, they are known to affect ECG preprocessing and diagnosis classification accuracy adversely. Therefore, they should be reduced before the circuit’s input, which amplifies the ECG. To reduce their effect, we aim to create a filter that reduces the high-frequency noise like EMG.

**Figure 2:**
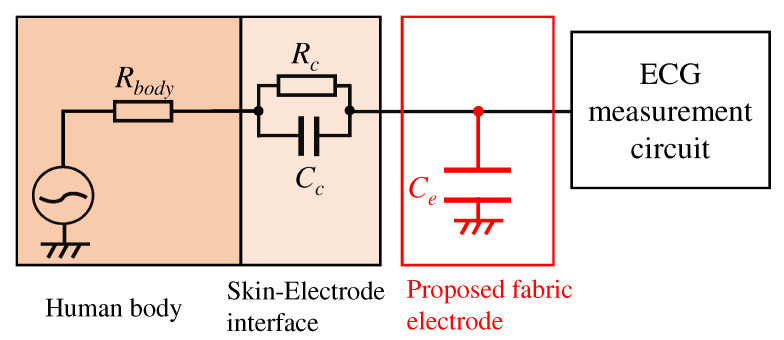
An overview of the proposed analog filter.

We first consider implementing a low-pass filter between the skin and electrode to attenuate the EMG. Figure 2 shows an electronic circuit of the low-pass filter. Elements constructed the low-pass filter in Figure 2 are $R_{body}$, $R_{c}$, $C_{c}$, and $C_{e}$. Each element means the internal resistance of human, the resistance, and the capacitor consist of skin-electrode impedance, and the capacitor of the proposed electrode, respectively. Here, we consider $R_{body}$ to be 500 Ω, which is the maximum internal resistance from the fingertip to the foot. It is known that the skin-electrode impedance consists of the resistor $R_{c}$ and capacitor $C_{c}$, which have several MΩ and several hundred pF to several nF, respectively [25]; however, the actual values of $R_{c}$ and $C_{c}$ are uncertain. If we can estimate the skin-electrode impedance, we can realize the low-pass filter by placing the appropriate values of $C_{e}$ before the circuit’s inputs. The transfer function combined with the $C_{e}$ can express as follows:

(1) $$\frac{V_{out}}{V_{in}}\left(s\right)=\frac{\frac{1}{sC_{e}}}{R_{body}+\frac{R_{c}}{1+sR_{c}C_{c}}+\frac{1}{sC_{e}}}.$$

Because the range of EMG is from 100 Hz to a few kHz or more, we measured the skin-electrode impedance to determine the appropriate capacitance of $C_{e}$.

### 3.2. Impedance Constituting the Textile Filter

**Figure 3:**
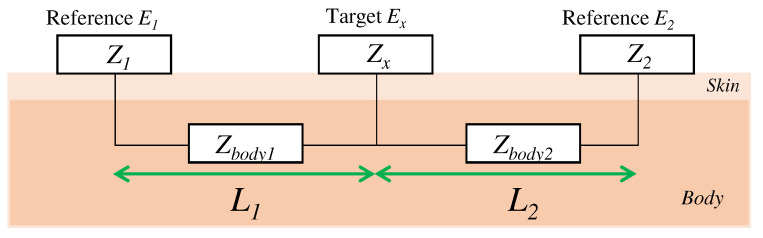
The relationship of the impedance between each electrode.

Here, we consider the skin-electrode impedance to determine the capacitance of the electrode to be designed. The skin-electrode impedance was measured by using one target electrode and two reference electrodes in accordance with previous work [19,26]. As shown in Figure 3, when a target electrode is attached between the reference electrodes, the impedance $Z_{1x}$ between the reference electrode $E_{1}$ and the target electrode $E_{x}$ can be measured. $Z_{1x}$ is the sum of the impedances $Z_{1}$, $Z_{body1}$, and $Z_{x}$, which are the impedance from $E_{1}$ to the skin, the impedance in the body, and the impedance from the skin to the target electrode, respectively. Similarly, the impedance $Z_{2x}$ from $E_{x}$ to $E_{2}$ and the impedance $Z_{12}$ from $E_{1}$ to $E_{2}$ are also the sum of the impedance of each electrode, the skin, and the impedance in the body. The following equation expresses the impedances between each electrode:

(2) $$\begin{array}{c} Z_{1x}=Z_{1}+Z_{body1}+Z_{x},\;\\ Z_{2x}=Z_{2}+Z_{body2}+Z_{x},\;\\ Z_{12}=Z_{1}+Z_{body1}+Z_{body2}+Z_{2}. \end{array}$$

Assuming that $Z_{body1}$ and $Z_{body2}$ are equal when the distances $L_{1}$ and $L_{2}$ from the target electrode $E_{x}$ to the reference electrodes $E_{1}$ and $E_{2}$ are equal, the final impedance $Z_{x}$ is expressed by the following equation:

(3) $$\begin{array}{c} \mathrm{if}\;L_{1}=L_{2}:\;\;\;\;\\ Z_{x}=Z_{1x}-\frac{Z_{12}}{2}=Z_{2x}-\frac{Z_{12}}{2}. \end{array}$$

**Figure 4:**
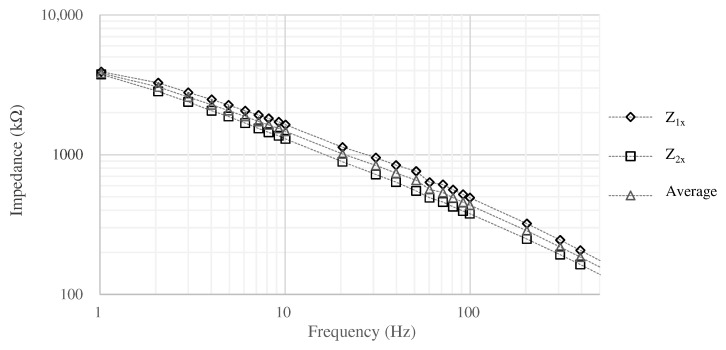
The impedance between the skin and the electrode.

In this case, impedance $Z_{x}$ was estimated based on Equation (3) by measuring $Z_{1x}$, $Z_{2x}$, and $Z_{12}$ when the surface area is 2×2$cm^{2}$. In the measurement, 3M Red Dot monitoring electrode was used as the reference electrode. The measurement results are shown in Figure 4. $Z_{12}$ was 151.2 kΩ at 1 Hz and 89.8 kΩ at 20 Hz, then we assume to be ignored when comparing with $Z_{1x}$ and $Z_{2x}$. The impedance above 10 Hz shows that the resistance and capacitance of the skin-electrode impedance are about 1.0 to 1.5 MΩ and about 1 to 3 nF, respectively. The measurement revealed the impedance between the skin and the electrodes, but in the actual environment, the impedance may change due to various factors (e.g., sweating). In order to determine the impedance of the electrodes that can be used as a filter even if the impedance of the skin changes, we conducted the simulation using the circuit shown in Figure 2 and LTspice.

**Figure 5:**
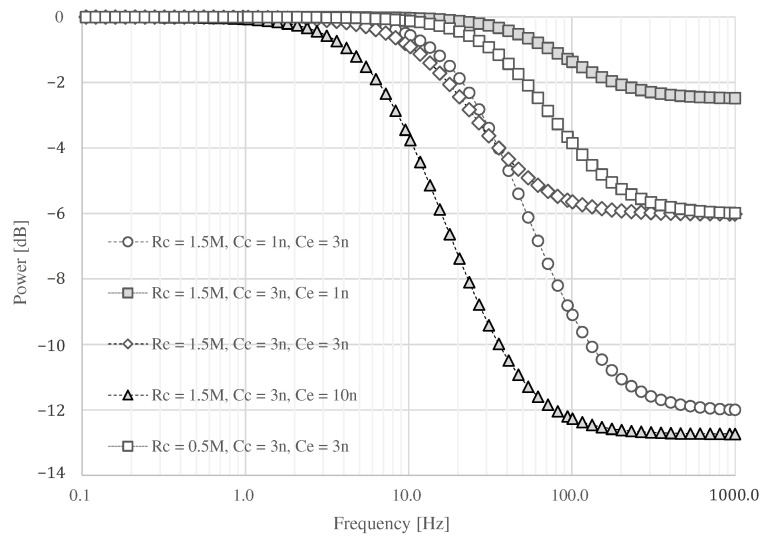
The simulation result changing $R_{c}$, $C_{c}$, and $C_{e}$.

The result of circuit simulation is shown in Figure 5. When $C_{c}$ is assumed to be 3 nF and $C_{e}$ is set to 1 nF, the attenuation above 100 Hz is as low as −2.48 dB at 1.0 kHz. On the other hand, an attenuation of about −6.02 dB can be expected by changing $C_{e}$ to 3 nF. If $C_{c}$ is 1 nF, a maximum attenuation of about −12.73 dB can be expected when $C_{e}$ is 3 nF. If the capacitance of $C_{e}$ is set to 10 nF or greater than $C_{c}$, it can be attenuated more than when $C_{e}$ is about 3 nF. However, the signal at about 10 Hz may be attenuated, and the ECG waveform may not be obtained. If the skin surface is wet, the resistance may decrease. If $R_{c}$ is set to 0.5 MΩ, the attenuated frequency moved into the high-frequency domain. Therefore, we assume that $R_{c}$ and $C_{c}$ are 1.5 MΩ and 3 nF, and we adopt 3 nF for $C_{e}$.

### 3.3. Fabric Structure

**Figure 6:**
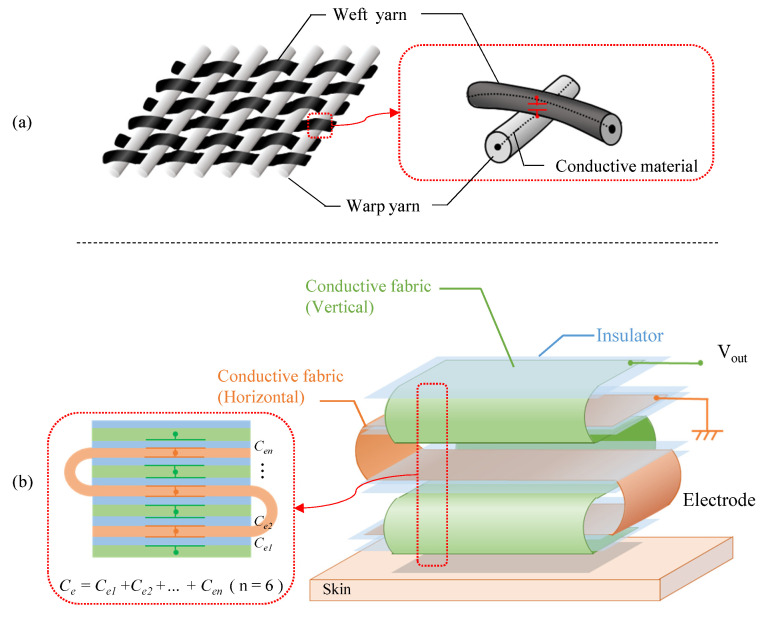
The structure of the fabric capacitor. (**a**) The conventional fabric such as pressure sensor. (**b**) The proposed fabric.

In this section, the actual structure of the electrode is described. As shown in Figure 6a, in the case of touch sensors made of conductive fabric, conductive yarns covered with an insulator, such as polyester, are crossed to create a capacitor. However, when the yarns are crossed, as in a pressure sensor with conductive yarns, the capacitance value per crossing point is at most several pF. To increase the capacitance value, we propose an electrode with the structure shown in Figure 6b.

**Figure 7:**
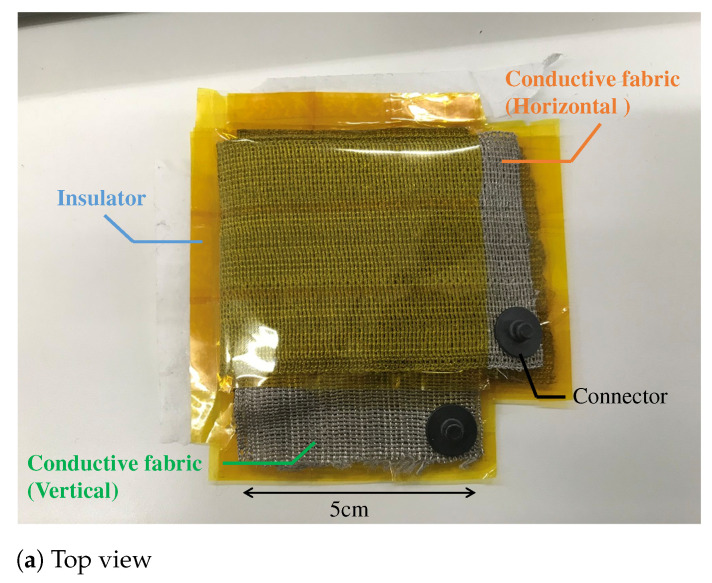

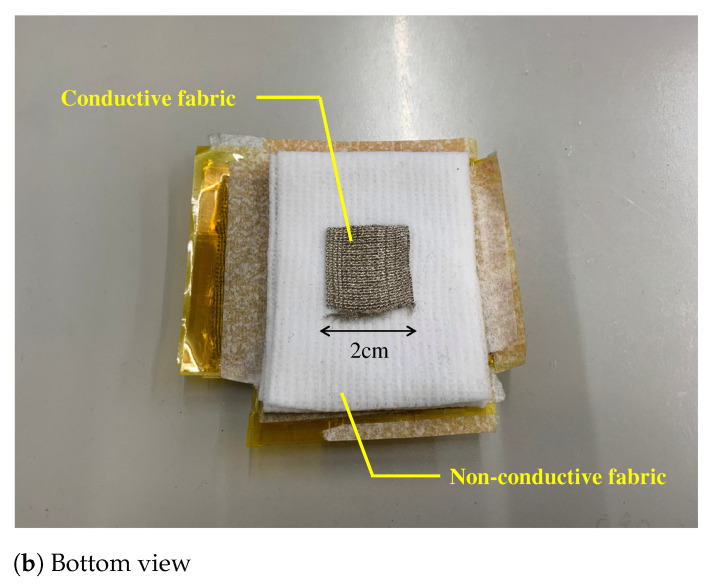
The prototype of the proposed electrode. (**a**,**b**) show the top view and bottom view, respectively. The fabric capacitor consists of the vertical and the horizontal conductive fabric. The area connecting with the skin is 2×2$cm^{2}$. To prevent skin touching the fabric capacitor, we used a non-conductive fabric in (**b**). The total thickness of the fabric electrode including that of a non-conductive fabric was about 1 cm.

The proposed electrode is made by sandwiching a non-conductive material between two pieces of conductive fabric and folding them. In focusing on the internal structure, the proposed structure is similar to the touch sensor in principle, because the sandwiched fabric layers work like a large number of capacitors in a fabric touch sensor. As a material of the proposed electrode, AGposs conductive wearable electrode tape was used. The tape used silver-plated conductive fibers, and the resistance per $cm^{2}$ was less than 1.0 Ω. In addition, silver has been reported to be less harmful to human health, making it a suitable material for electrodes that are directly attached to the human body. A polyimide was used as the non-conductive material to stack conductive tape. Although we have not evaluated the flexibility of the filter, we confirmed that it could be attached to the arm even if the ground surface was curved, and that it was flexible enough not to impair its function as a filter in actual measurements. The capacitance per $cm^{2}$ when the fabric tape stacked with the polyimide was measured with a LCR Meater, and it was 20 pF. Therefore, the capacitor must have a surface area of about 150 $cm^{2}$ due to the capacitance per unit area. As shown in Figure 7, the area between the skin and electrode was 2×2 $cm^{2}$. The capacitor was made from the conductive tape with a length of 30 cm and a width of 5 cm. The electrode has seven layers, and the capacitance of each layer can be regarded as a single capacitor that is synthesized.

**Figure 8:**
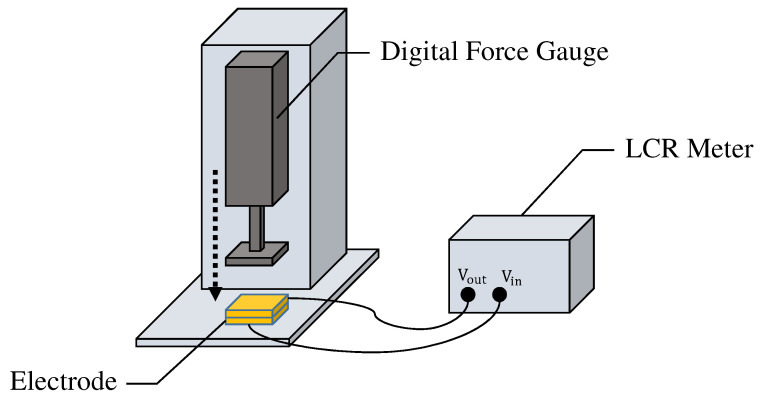
The environment for measuring the capacitance of the electrode.

It should be noted that the capacitance of the fabric filter changes according to the applied pressure due to the characteristics of folded fabric. We further investigated the differences in the capacitance of two prototype electrodes when different external forces are applied to them. As shown in Figure 8, a digital force gauge and LCR meter were used to measure the capacitance by applying constant pressure to the electrodes. The capacitance of each electrode was measured 600 times every 0.5 seconds for about five minutes. The average values of the capacitance when 10, 30, and 50 N were applied to one of the electrodes were 1.66, 2.24, and 3.35 nF, respectively. Similarly, the average values for the other electrode were 2.39, 2.96, and 3.34 nF, respectively. There was a difference in capacitance of about 0.75 nF when an external force of 10 N was applied to each electrode, but the capacitance became almost the same value when the external force was increased. Therefore, once the pressure is applied, the capacitance value in the fabrics can be kept almost constant.

## 4. Evaluation of Textile Filter

In Section 3, it was confirmed that the proposed capacitor was determined to have the desired capacitance. We evaluated whether it can be used as an electrode of an ECG measurement device through an actual measurement. The signal quality obtained by our proposed electrodes and ordinal Ag/AgCl ones was compared.

### 4.1. Settings

**Figure 9:**
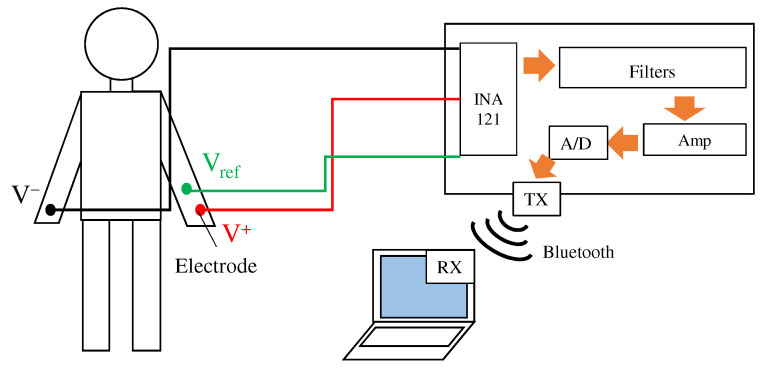
Overview of the ECG measurement system. The system has three inputs from the right arm $V^{-}$, the left arm $V^{+}$, and the reference electrode $V_{\mathrm{ref}}$. $V^{-}$ and $V^{+}$ are our proposed fabric electrodes, and $V_{\mathrm{ref}}$ is a non-folded fabric electrode. The ECG signal is sent to laptop via Bluetooth.

Because the fabric filter was connected before the pre-amplifier, it was necessary to confirm the attenuation of the noise by paying attention to the noise mixed with the movement of the body. Therefore, ECGs at rest and exercise were performed. During exercise, the subject grasped the grip strength meter to mix the EMG; in order to confirm the attenuation of the EMG mixed in when grasping an object, a wristband-type system that affects the EMG more was used. Figure 9 shows an overview of the system used for the ECG measurement. As in the conventional method, this system acquires the ECG using electrodes and electronic circuits attached to the body. The acquired ECG signals are converted into a digital signal by a 12-bit A/D converter in ESP32 and are then stored in a paired terminal through the Bluetooth module. The sampling frequency during the measurement was set to 1.0 kHz. System power was supplied from a mobile battery to minimize noise generated from the GND of the measurement equipment.

**Figure 10:**
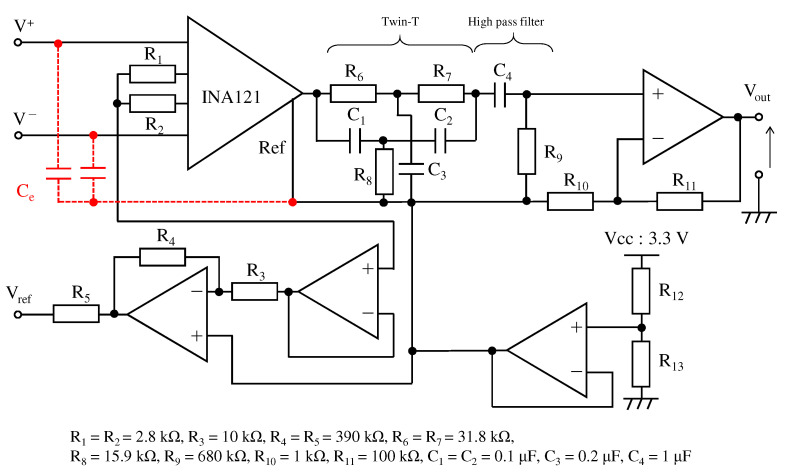
The circuit for the ECG measurement. The red line indicates the proposed electrode. The circuit consists of the twin-t notch filter, the first order high-pass filter and the amplifiers.

Figure 10 shows the electronic circuit used in the ECG measurement system. The signal obtained from the right-hand and left-hand electrodes is input to the instrumentation amplifier INA121 (Texas Instruments, input impedance 1 TΩ). The first stage instrumentation amplifier and the second stage non-inverting amplification circuit amplified 20 dB and 40 dB, respectively, resulting in a total amplification of 60 dB. Because the aim was to confirm the performance of a low-pass filter using the textile filter, the filters between the first-stage amplification and the second-stage amplification were combined as a twin-t notch filter and a high-pass filter. The twin-t notch filter is composed of a resistor and a capacitor, and it reduces noise from a commercial power supply of 50 Hz. The cut-off frequency of the high-pass filter was set to 0.5 Hz. To confirm the effect of noise reduction on the high frequency of the proposed fabric electrode, we experimented with the proposed electrode and fabric electrode without the proposed structure called non-folded fabric. The non-folded fabric electrode also has a 2×2 $cm^{2}$ surface area and was connected to the input of the electronic circuit directly.

For the evaluation of the acquired signal, signal-to-noise ratio (SNR) was used as a quantitative evaluation index. The SNR calculation is as follows:

(4) $$\begin{array}{c} SNR=10\log_{10}\frac{\sum_{x=1}^{N}S_{x}^{2}}{\sum_{x=1}^{N}{\left(S_{x}-S_{x}^{\prime }\right)}^{2}}, \end{array}$$

where $S_{x}$ and $S_{x}^{\prime }$ denote the x-th data of the clean signal and the acquired signal, respectively. To evaluate the difference of the high-frequency component, we applied the FIR filters to $S_{x}$. These filters are the high-pass filter ($f_{c}$ = 0.67 Hz), the bandwidth rejection filter ($f_{c}$ = 49 Hz, 51 Hz), and the low-pass filter ($f_{c}$ = 100 Hz). Similarly, the same high-pass filter and bandwidth rejection filter was applied to $S_{x}^{\prime }$. In addition, power spectral density (PSD) is also shown to indicate the frequency components contained in the measured signals. The method of Welch et al. [27] was used to calculate the PSD. To further compare the morphology of the ECG, we used temporal and spectral measures of HRV, peak-to-peak amplitude, and cross-correlation index to compare with Ag/AgCl electrodes, similar to the indices described in [12]. The ECG measurements using the proposed electrode and the non-folded fabric electrode were taken at rest and during exercise for three minutes, once for each. The subject was a university male student in his 20 s. In order to check the feasibility of using proposed fabric electrodes, the experiment was conducted with only one subject.

### 4.2. The Measurement Result during Resting

**Figure 11:**
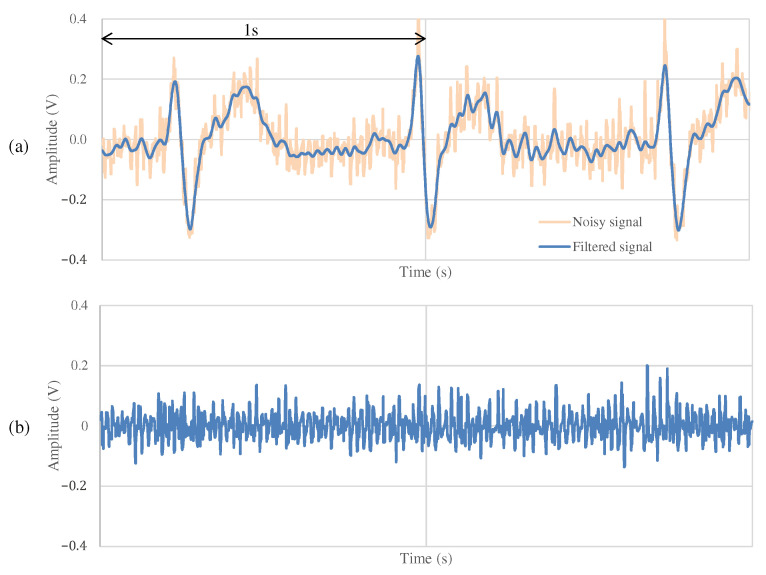
The ECG measurement result using the non-folded fabric electrode at rest. (**a**) The measured signal and the signal filtered by FIR filters. (**b**) The high-frequency component above 100 Hz in (**a**). (**a**,**b**) are the common in Figure 12, Figure 13 and Figure 14.

**Figure 12:**
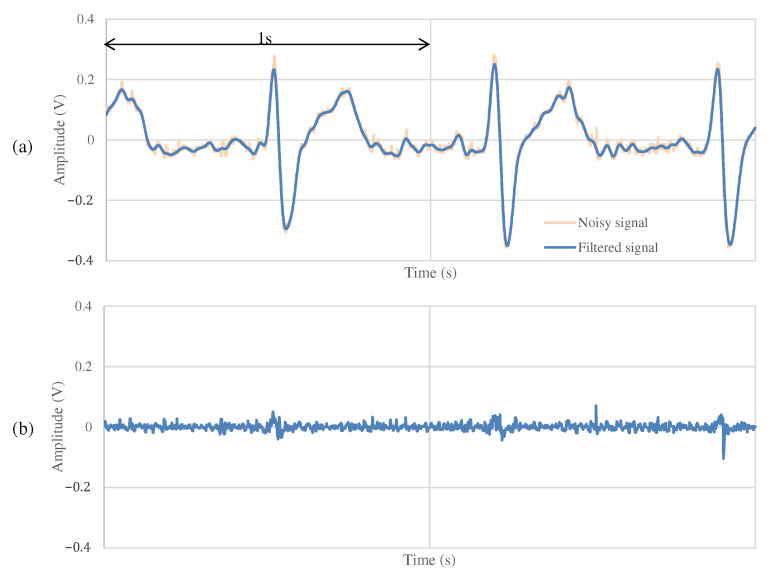
The ECG measurement result using the proposed fabric electrode at rest.

Figure 11 and Figure 12 show the ECG measurement results of the non-folded fabric electrode and the proposed electrode, respectively. The signal using a non-folded fabric electrode in Figure 11a is noisy, and it can be seen that the R-peak is buried in the noise. In particular, the potential of the T wave after the R-peak is similar to the R-peak, making it difficult to distinguish between the R wave and the T wave. The signal obtained from the proposed electrode in Figure 12a has less noise than in Figure 11a, and it is easier to differentiate between R and T waves. Compared to Figure 12b, Figure 11b has more high-frequency component other than ECG. SNR values using the non-folded fabric and proposed fabric based on Equation (4) were 7.43 and 19.45 dB, respectively. It means that the SNR value was improved 12.02 dB by using the proposed electrode. Therefore, we can confirm the attenuation of the high-frequency noise by the filtering of the proposed electrode.

### 4.3. The Measurement Results during Exercise

**Figure 13:**
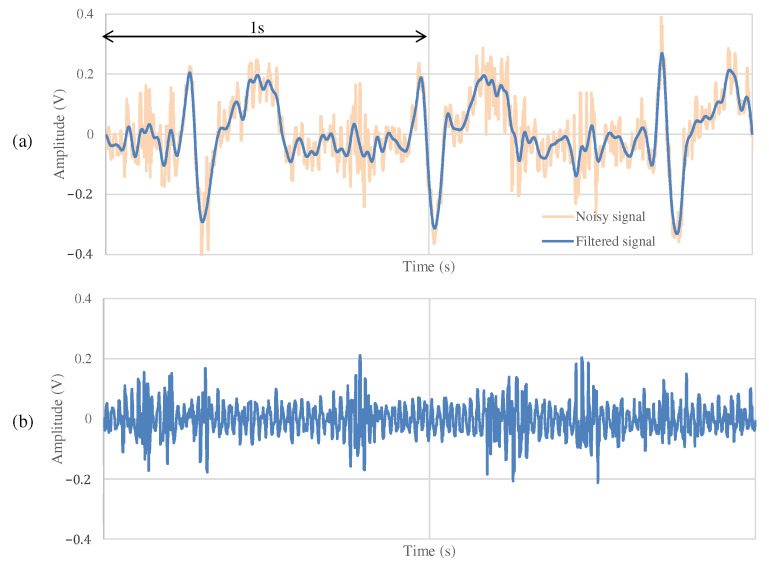
The ECG measurement result using the non-folded fabric electrode in exercise.

**Figure 14:**
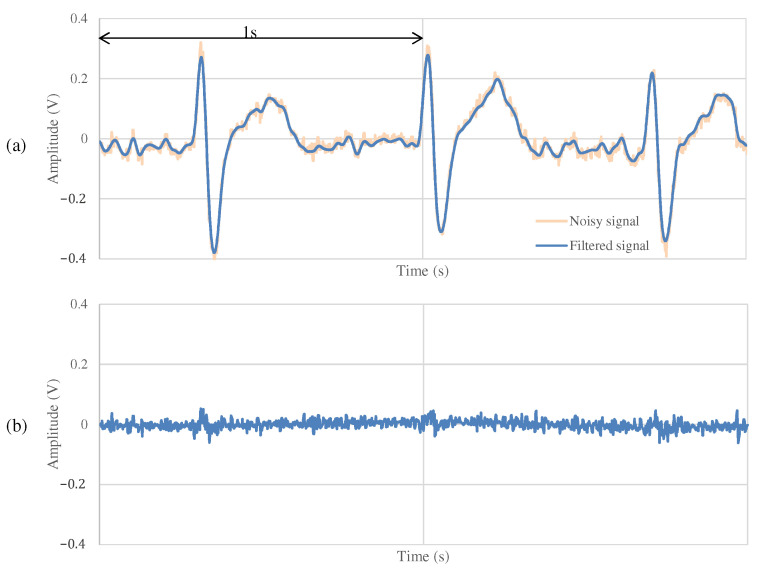
The ECG measurement result using the proposed fabric electrode in exercise.

The results of the measurements during exercise using the non-folded fabric electrode and the proposed electrode are shown in Figure 13 and Figure 14, respectively. As shown in Figure 13a, the signal measured during exercise had more noticeable noise overall than that in Figure 11a. Similarly, when using the proposed electrode, the signal in Figure 14a contains more noise than in Figure 12a. As shown in Figure 13b, the high-frequency noise other than ECG contained in Figure 13a is greater than the noise in Figure 11b. Therefore, we can see that this signal is the noise introduced by the exercise. On the other hand, as shown in Figure 14b, the signal using the proposed electrode has less noise than that in Figure 12b. SNR values using the non-folded fabric and proposed fabric based on Equation (4) were 7.59 and 17.88 dB, respectively. As well as during resting, the SNR value in exercise was also improved 10.29 dB by using the proposed electrode. Therefore, it can be said that high-frequency component noise can be reduced by incorporating a filter structure in the electrodes, even during exercise.

**Figure 15:**
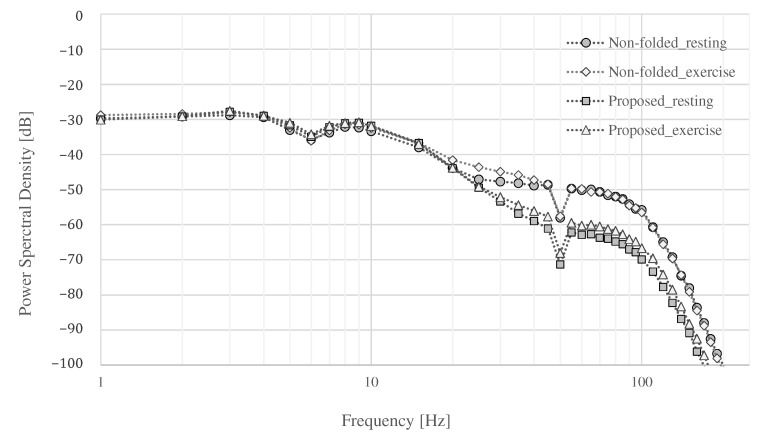
The power spectral density.

Finally, the power spectral density (PSD) of each measurement is shown in Figure 15. The power of all signals was similar up to around 20 Hz, and the power of the signals above 20 Hz differed depending on whether they were at rest or exercise, and whether the proposed electrode or non-folded fabric electrode was used. Both electrodes showed an increase in power above 100 Hz during exercise. An attenuation of −6.02 dB was expected in the simulation result, as shown in Figure 5, where $R_{c}$, $C_{c}$, and $C_{e}$ were assumed to be 1.5 MΩ, 3 nF, and 3 nF, respectively. The attenuation above 100 Hz by the proposed electrode was −5.96 to −10.56 dB at rest and −6.67 to −10.91 dB during exercise compared with the ordinal electrodes. The average attenuation above 100 Hz were −9.06 dB at rest and −9.41 dB during exercise compared with the ordinal electrodes. Therefore, the performance of the low-pass filter with the proposed electrode was confirmed as desired.

### 4.4. Signal Quality Comparison with Ag/AgCl Electrode

**Figure 16:**
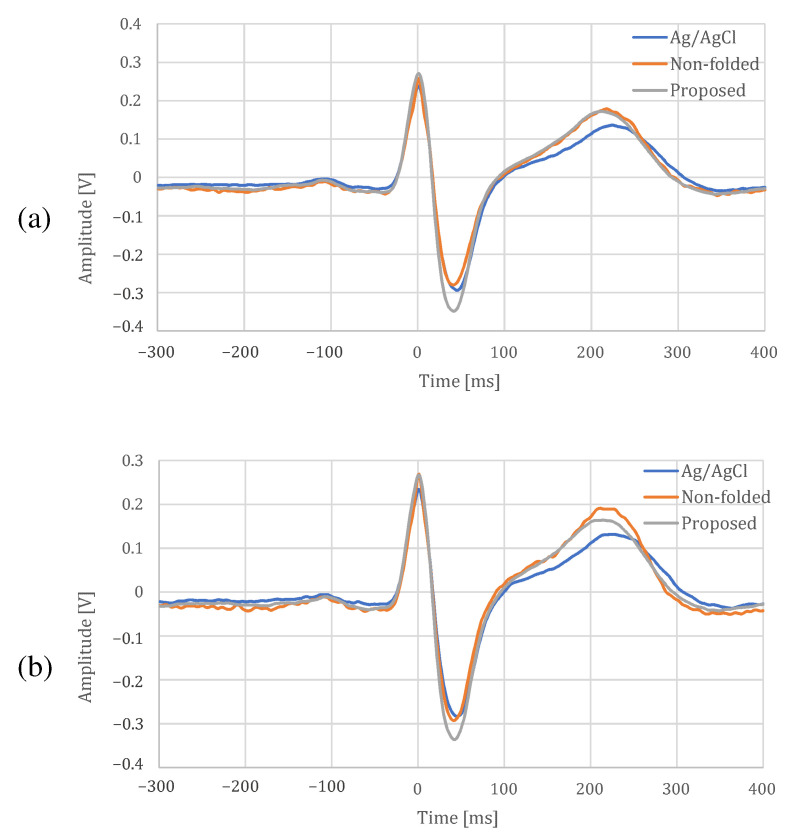
ECG template for each electrode. (**a**) At rest, (**b**) during exercise.

The comparison between the proposed electrode and an Ag/AgCl electrode at rest and during exercise is shown in Figure 16, Table 1 and Table 2. In Figure 16, there is a difference in the shape of T-wave between the fabric electrode and an Ag/AgCl electrode, but the other shapes are similar. In addition, it can be confirmed that the proposed electrode can reduce the noise because distortions can be seen on the non-folded electrode compared with the proposed electrode. In Table 1 and Table 2, the cross-correlation coefficients were slightly larger for the proposed electrode than for the non-folded fabric, and the proposed electrodes was closer to the Ag/AgCl results. All the temporal measures were lower for the proposed electrode in both resting and exercise conditions. The larger NN50 values for the Ag/AgCl and the non-folded electrodes were due to the fact that the function of low-pass filter was not installed in both of them, and only the proposed electrode with a built-in low-pass filter was more stable. In spectral measures, the power of the Ag/AgCl electrode was the highest, and that of the proposed electrode was the lowest. The peak-to-peak amplitude of the proposed electrode was highest, and the differences from the Ag/AgCl electrode were 0.081 V at rest and 0.086 V during exercise. From the experimental results above, we confirmed that the proposed electrode can measure ECG, as well as an Ag/AgCl electrode.

## 5. Discussion

The experimental results showed that the proposed fabric electrodes attenuated the high-frequency component both at rest and exercise. Due to the characteristics of the analog filter embedded in the electrode, there was a possibility that the phase of each electrode would be shifted and the ECG could not be observed, but the ECG was confirmed with the proposed electrode. It was shown that the fabric itself can be used as a passive device by changing the internal structure of the fabric electrodes, and it can be applied as an analog filter. The filter characteristics can be adjusted by the area and the number of layers, so it can be used as an equalizer. Since the capacitance can be changed for each location on a plane, it may be used in conjunction with signal analysis to obtain signals with less noise, and also to analyze signals that vary from location to location. For more efficient noise attenuation, we believe there are improvements that can be made in terms of electronic circuits and electrodes.

To confirm the performance of the fabric electrode filter, we compared the minimum electronic circuits required to obtain the ECG. However, in the actual ECG measurement system, it is also possible to combine an analog filter with the fabric electrode and a low-pass filter using more passive devices like chip capacitors and resistors. The improvement of noise reduction performance combining the fabric electrodes and the electronic circuit is an issue to be addressed in the future. In addition, because the proposed fabric electrode used a low-pass filter using the impedance of the human body, the attenuation of the high-frequency component did not decrease after a certain value. Therefore, we consider it possible to extend it to a multi-stage filter using only conductive fabric by combining fabric materials that can be used in place of resistors and capacitors. If a multi-stage filter such as a band-pass filter could be created, it would be possible to design it with a more stable frequency response independent of the pressure applied to the skin and electrode. In the future, we will focus on the fine structure at the thread level and study the electrode structure that can be incorporated into a fabric electrode as a passive device. In the experiment, the capacitance of fabric electrode $C_{e}$ was stable when it was sufficiently pressed with a band, although the layers of the proposed electrode were not bonded to each other. It will be possible to make the electrode more stable by replacing the layers with a woven structure. The structure of weaving coated conductive yarns is promising for more capacity and reduced thickness and area. Capacitance is generated between the yarns, then capacitance is generated even within a single piece of fabric. Furthermore, by weaving the fabric into layers, we can realize even more capacitance. Additionally, it is possible to replace the material with a more flexible one, and it is desirable to discover such a material.

## 6. Conclusions

In this paper, we proposed a fabric electrode in which the fabric itself plays the role of a filter without the conventional passive devices, such as chip capacitors and resistors. The proposed electrode has sufficient capacitance to form an analog filter by folding the conductive fabric. By combining the capacitor with the skin-electrode impedance, the electrode has the function of a low-pass filter, which attenuates high-frequency component noise. During the evaluation, the ECG was performed while the subject was at rest and during exercise while being intentionally influenced by EMG noises. It was confirmed that the SNR values of the proposed electrode improved around 12.02 dB at rest and 10.29 dB during exercise compared to when the non-folded fabric electrode was used. The results indicate that the performance of the folded fabric capacitor is comparable to a typical ceramic capacitor and that fabric electrodes can be used for ECG measurements. $R_{c}$ and $C_{c}$ in the skin-electrode interface can differ when the skin is sweating or the electrode is wet. We have confirmed that the desired filter characteristics can be obtained within a certain range, as shown in Figure 5. However, it is necessary to consider the fact that the filter characteristics may not be within this range during intense exercise. We believe that there are enough applications where the electrode can be used despite the limitations. In future work, we will clarify the optimal capacitor parameters and the structure of the new fabric electrode. Additionally, we would like to verify the versatility of the system by increasing the number of subjects and conducting experiments over a long period of time as future work.

## Figures and Tables

**Table 1:** ECG signal quality indices at rest.

	Parameter	Ag/AgCl	Non-Folded	Proposed
Cross-correlation index	ρ (unitless)	—	0.980	0.988
Temporal measures of HRV	meanNN(ms)	729.47	740.29	701.88
	SDNN(ms)	50.352	33.434	27.941
	RMSSD(ms)	31.712	30.151	20.256
	NN50(ms)	25	20	2
Spectral measures of HRV	LF (${\mathrm{ms}}^{2}$)	367.27	259.33	291.41
	HF (${\mathrm{ms}}^{2}$)	527.88	188.06	124.63
	Total (${\mathrm{ms}}^{2}$)	1151.20	628.23	498.12
	LF/HF (unitless)	0.696	1.379	2.338
Peak-to-peak amplitude	$V_{\mathrm{pp}}$ (V)	0.545 ± 0.040	0.587 ± 0.050	0.626 ± 0.042

**Table 2:** ECG signal quality indices during exercise.

	Parameter	Ag/AgCl	Non-Folded	Proposed
Cross-correlation index	ρ (unitless)	—	0.974	0.988
Temporal measures of HRV	meanNN(ms)	716.47	722.22	684.02
	SDNN(ms)	42.514	33.083	27.719
	RMSSD(ms)	32.563	29.722	26.176
	NN50(ms)	29	24	17
Spectral measures of HRV	LF (${\mathrm{ms}}^{2}$)	384.62	193.73	123.57
	HF (${\mathrm{ms}}^{2}$)	324.55	212.31	126.47
	Total (${\mathrm{ms}}^{2}$)	1010.30	518.33	298.14
	LF/HF (unitless)	1.185	0.913	0.977
Peak-to-peak amplitude	$V_{\mathrm{pp}}$ (V)	0.541 ± 0.043	0.625 ± 0.068	0.627 ± 0.047

## Data Availability

The data presented in this study are available on request from the corresponding author.
